# Repeated epidural delivery of Shinbaro2: effects on neural recovery, inflammation, and pain modulation in a rat model of lumbar spinal stenosis

**DOI:** 10.3389/fphar.2024.1324251

**Published:** 2024-05-17

**Authors:** Jin Young Hong, Changhwan Yeo, Hyun Kim, Junseon Lee, Wan-Jin Jeon, Yoon Jae Lee, In-Hyuk Ha

**Affiliations:** Jaseng Spine and Joint Research Institute, Jaseng Medical Foundation, Seoul, Republic of Korea

**Keywords:** epidural injection, Shinbaro2, lumbar spinal stenosis, neuropathic pain, pain relief, inflammation

## Abstract

The choice of treatment for lumbar spinal stenosis (LSS) depends on symptom severity. When severe motor issues with urinary dysfunction are not present, conservative treatment is often considered to be the priority. One such conservative treatment is epidural injection, which is effective in alleviating inflammation and the pain caused by LSS-affected nerves. In this study, Shinbaro2 (Sh2), pharmacopuncture using natural herbal medicines for patients with disc diseases, is introduced as an epidural to treat LSS in a rat model. The treatment of primary sensory neurons from the rats’ dorsal root ganglion (DRG) neurons with Sh2 at various concentrations (0.5, 1, and 2 mg/mL) was found to be safe and non-toxic. Furthermore, it remarkably stimulated axonal outgrowth even under H_2_O_2_-treated conditions, indicating its potential for stimulating nerve regeneration. When LSS rats received epidural injections of two different concentrations of Sh2 (1 and 2 mg/kg) once daily for 4 weeks, a significant reduction was seen in ED1^+^ macrophages surrounding the silicone block used for LSS induction. Moreover, epidural injection of Sh2 in the DRG led to a significant suppression of pain-related factors. Notably, Sh2 treatment resulted in improved locomotor recovery, as evaluated by the Basso, Beattie, and Bresnahan scale and the horizontal ladder test. Additionally, hind paw hypersensitivity, assessed using the Von Frey test, was reduced, and normal gait was restored. Our findings demonstrate that epidural Sh2 injection not only reduced inflammation but also improved locomotor function and pain in LSS model rats. Thus, Sh2 delivery via epidural injection has potential as an effective treatment option for LSS.

## 1 Introduction

Lumbar spinal stenosis (LSS) occurs upon narrowing of the spinal canal, the protective conduit for nerves and blood vessels, leading to pressure on the spinal cord and surrounding nerves, thus compromising patient wellbeing (Siebert et al., 2009; Genevay and Atlas, 2010). The symptoms of LSS of the lower spine intensify with age, with increasing pain, weakness, and restricted mobility (Lurie and Tomkins-Lane, 2016; Lee et al., 2020). For mild to moderate symptoms, conservative treatment emphasizing non-invasive methods is recommended for palliative care and enhancing mobility (Gunzburg and Szpalski, 2003; Fornari et al., 2020). Common approaches include physical therapy to improve spine flexibility and strength, medications like non-steroidal anti-inflammatory drugs to reduce pain and inflammation, epidural steroid injections for temporary relief, lifestyle modifications such as posture improvement and weight management, and alternative therapies like acupuncture or chiropractic care. Treatments are often combined and tailored to the patient’s specific needs (Gunzburg and Szpalski, 2003; Oka et al., 2018).

Epidural injections are considered a better alternative for cases that fail to respond to conservative treatments (Ghai et al., 2013; Yang et al., 2020). Direct epidural administration in patients with disc diseases is primarily aimed at managing pain and inflammation. Typically, these injections include steroids to reduce inflammation around impacted nerve roots and provide prompt relief from pain and discomfort (Cohen et al., 2013). This immediate relief often reinvigorates patients, facilitating a swift return to daily activities. However, the therapeutic effects of steroids are transient, necessitating periodic reinjections (Carassiti et al., 2021). While effective, the recurrent use of epidural steroids is not without drawbacks, with side effects ranging from transient issues like elevated blood pressure and mood fluctuations to rarer, more severe complications like nerve damage (Stafford et al., 2007; Bellini and Barbieri, 2013). Furthermore, being palliative in nature, steroids offer symptomatic relief but do not address the root causes of the disc ailments (Liu et al., 2015). This limitation of steroidal injections has driven a quest for alternative epidural injectables that address the root causes of LSS.

In this study, we evaluated the therapeutic efficacy of the epidural application of Shinbaro2 (Sh2), a pharmacopuncture solution derived from GCSB-5 (Cheongpa-jeon), in a rat model of LSS. GCSB-5 is a composite formulation containing a blend of six distinct wild herbs, including *Cibotium barometz, Bang-Poong (Saposhnikovia divaricata [Turcz] Schischkin)*, *Eucommia ulmoides*, and *Ogapi* (*Acanthopanax sessiliflorum*, *Achyranthes japonica*, and *Glycine max*) (Namgoong et al., 2022). This formulation has undergone standardization by the Korea Food and Drug Administration (KFDA), and is renowned for its multifaceted therapeutic properties, including anti-inflammatory, neuroprotective, cartilage regeneration, and analgesic effects (Kim et al., 2011; Kim et al., 2012; Ha et al., 2016). Sh2 is a modified version of GCSB-5, whose effectiveness is further enhanced by the addition of four supplementary herbs: *Ostericum koreanum*, *Angelica pubescens*, *Paeonia albiflora*, and *Scolopendra subspinipes*. This combination offers a unique and potentially more efficacious pharmacopuncture solution for various musculoskeletal therapeutic applications (Lee et al., 2017; Hong et al., 2023a; Kim et al., 2023).

Clinically, Sh2 has been commonly used as a non-surgical remedy for spinal stenosis in Korea (Lee et al., 2017). In animal models, whether delivered intramuscularly or orally, Sh2 has shown promising results in functional recovery due to its anti-inflammatory and analgesic properties (Park et al., 2019; Kim et al., 2023). This study aimed to assess the sustained benefits of Sh2 when administered epidurally over extended durations. We further explored the feasibility of its clinical application through the epidural route and sought to define optimal concentration ranges.

## 2 Materials and methods

### 2.1 Preparation of Sh2

The Sh2 herbal acupuncture solution is derived from a selection of nine dried wild herbs, each possessing unique properties and therapeutic potential. The nine herbs included *C. barometz* rhizome (0.0013 g/mL, txid29588), the root of *Saposhnikovia divaricate* (0.0013, txid203717), the stem bark of *E. ulmoides* (0.0013, txid4392), the stem and root of *Acanthopanax sessiliflorus* (0.0013, txid105886), rhizomes and roots of *O. koreanum* (0.0013, txid182415), the root of *A. pubescens* (0.0013, txid312530), the root of *A. japonica* (0.0013, txid543011), *S. subspinipes* (0.0013, txid55038), and the root of *P. albiflora* (0.0027, txid35924)]. To prepare the injectable Sh2, herbs are thoroughly boiled in 70% ethanol for 3 h to effectively extract their active components. After boiling, the extract is freeze-dried into a powder form, preserving the medicinal properties of the herbs and enhancing the convenience of use. This powder then undergoes a sterile filtration process in compliance with Good Manufacturing Practice (GMP) standards. Sterilization includes primary and secondary filtration through a 0.2 µm filter under clean nitrogen pressure. Additionally, the integrity of the sterilizing filters is rigorously tested before and after use to ensure their effectiveness and safety. The extracted solution is quantified and adjusted with injection water (for pharmacopuncture) by adding pH adjusters and additives such as NaCl to achieve a pH of 7.0–7.4 and a salinity of 0.9%. Filling sets, rubber stoppers, vials, and aluminum caps are cleaned and sterilized before being brought into the sterile room. The pharmaceutical preparation is sterile filtered and aliquoted into vials in volumes greater than 2.0 mL each, followed by stoppering with rubber stoppers and sealing with aluminum caps. The process involves supplying the chamber with high pressure and conducting a high-pressure steam sterilization process. After sterilization, each vial is inspected for foreign materials, and random samples are tested for insoluble particles. Microbiological and endotoxin testing, among other specified quality control assessments, are conducted. Vials that pass these tests are labeled and packaged for storage at low temperatures. In this manner, the Sh2 herbal acupuncture solution (2 mg/mL) is prepared and supplied by Jaseng Oriental Medicine Hospital. It is then diluted with injection water for epidural administration to LSS rats, achieving concentrations of one or 2 mg/kg in a volume of 100 μL for epidural injection.

### 2.2 Primary rat dorsal root ganglion neuron extraction

The primary culture of neurons from dorsal root ganglion (DRG) neurons was carried out using an adapted version of the method described by Kim et al. (Kim et al., 2016). DRGs were harvested from young adult Sprague–Dawley (SD) rats (Daehan Bio Link, Chungju, Korea; aged 5–6 weeks; Approval No. JSR-2023-02-002-A, Jaseng Animal Care and Use Committee). The rats were euthanized via CO_2_ inhalation and perfused upon injecting approximately 20 mL of cold Hank’s balanced salt solution (HBSS; Gibco BRL, Grand Island, NY, United States) and 100 μL of 2% heparin (Joongwae Pharma Corporation, Seoul, Korea) into the left ventricle. The right atrium was then incised for cardio-perfusion; vertebrae from C2 to L7 were swiftly subjected to laminectomy to expose DRGs, which were then placed in chilled Petri dishes. Using No. Five fine forceps and microscissors under a microscope, the DRGs were isolated and gathered in a Petri dish with cold HBSS. The DRGs were further desheathed in HBSS under high magnification.

Next, DRGs underwent enzymatic digestion using 40 U/mL papain in F12 medium (Gibco BRL) containing 1% GlutaMAX (Gibco BRL) at 37°C for 10 min. The digested samples were centrifuged at 180 *g* for 1 min, and the supernatants were discarded. The cell pellet was treated with 4 mg/mL collagenase (Type II, Worthington Biochemical, Lakewood, NJ) and 4.66 mg/mL dispase (Type II, Roche Applied Sciences, Mannheim, Germany) at 37°C for 40 min. Post-centrifugation at 180 *g* for 1 min, supernatants were discarded, and cells were washed with 2 mL Neurobasal medium supplemented with B27, GlutaMAX, 1% penicillin/streptomycin (all from Gibco-BRL), and 10 ng/mL recombinant brain-derived neurotrophic factor (PeproTech, Rocky Hill, CT, United States). After the DRG tissues settled, they were gently triturated approximately 10–12 times in 500 μL of culture medium. After filtering through a 70-μm strainer to eliminate debris, the dissociated cells were placed on uncoated culture dishes and incubated in a CO_2_ incubator for 1 h. Finally, the DRG neurons were transferred to plates coated with 20 μg/mL poly-D-lysine and 10 μg/mL laminin (both supplied by Gibco-BRL).

### 2.3 hydrogen peroxide and Sh2 treatment

We adapted the stabilization period, treatment timing, and duration for H_2_O_2_ and Sh2 on primary cultured DRG neurons from our previous research designs, incorporating slight modifications based on literature that examined neurite length under various DRG culture periods (Lee et al., 2017; Hong et al., 2023a). Following seeding in 6/12/24-well plates, the cells were cultured for 48 h before a 30 min pretreatment with 50 μM H_2_O_2_. Subsequently, three varying Sh2 concentrations (0.5, 1, and 2 mg/mL) were introduced to the cell culture medium. Over the next 24 h, cells were incubated in the presence of H_2_O_2_ and Sh2. Post-incubation, samples were fixed using 4% paraformaldehyde (PFA; Biosesang, Seongnam, Korea) for later assessment.

### 2.4 CCK assays

The Cell Counting Kit-8 assay (CCK-8; Dojindo, Kumamoto, Japan) was employed to gauge cell viability, as described previously (Hong et al., 2022). In brief, DRG neurons were arranged in 96-well plates. To assess Sh2-induced neurotoxicity, we performed serial dilutions, halving the concentration starting from the original solution, to establish a range of concentrations from 0.0625 to 2 mg/mL for experimental screening. The neurons were then exposed to these varying Sh2 concentrations (0.0625, 0.125, 0.25, 0.5, 1, and 2 mg/mL). After incubation according to the set schedule, CCK-8 solution was added to the cells (10% of the total volume) and incubated for 4 h. The absorbance at 450 nm was noted using a microplate reader (BioTek Epoch Microplate Reader).

### 2.5 Cell viability assays and imaging

To evaluate cell viability following H_2_O_2_ exposure and Sh2 treatment, we employed a live/dead cell imaging assay (Invitrogen, Grand Island, NY, United States). This assay utilizes two dyes: calcein AM, which stains live cells green, and BOBO-3 iodide (EthD-1), to distinguish between live and dead cells. Post-treatment, DRG neurons were incubated with the assay’s fluorescent probes at 37°C for 15 min. Subsequent images were acquired at ×100 magnification using a confocal microscope (Eclipse C2 Plus; Nikon, Minato City, Tokyo, Japan). All imaging parameters, such as laser power, gain value, magnification, and scanning time, were uniformly maintained across all groups during capture. ImageJ software (version 1.37, NIH, Bethesda, MD, United States) was used to analyze the intensities of green (live) and red (dead) cells from ten randomly selected images without employing any additional measurement options.

### 2.6 Immunocytochemistry

Cell fixation, permeabilization, blocking, and immunocytochemistry were performed according to previously reported methods (Kim et al., 2017). Primary cultured DRGs were fixed using 4% PFA (Biosesang) for 30 min, followed by three 5-min washes in phosphate-buffered saline (PBS) (Gibco BRL). The cells were permeabilized with 0.2% Triton X-100 for 10 min and blocked using 2% normal goat serum for 1 h. Primary antibodies, including anti-Tuj1, anti-NeuN, anti-TRPV1, anti-NF200, IB4, and anti-CGRP, were added to the cells and incubated overnight at 4°C. Cells were then exposed to fluorescein isothiocyanate-conjugated secondary antibodies (Jackson ImmunoResearch Laboratories, West Grove, PA, United States) for 2 h. After three 5-min washes with PBS, the cells were processed with fluorescence mounting medium (Dako Cytomation, Carpinteria, CA, United States). Images were captured at 100 × or × 400 magnification using a confocal microscope (Eclipse C2 Plus; Nikon).

### 2.7 LSS surgery and epidural Sh2 injection

Male SD rats (7 weeks of age; 230–250 g) were chosen for the *in vivo* experiments, adhering to guidelines from the Jaseng Animal Care and Use Committee (Approval No. JSR-2023-01-005-A). Animals were housed at a constant temperature of 24°C ± 1°C, with a relative humidity between 45% and 55%, and subjected to a 12:12-h dark/light cycle. They had unlimited access to food and water. The maintenance and experimental procedures complied with the AVMA Guidelines for the Euthanasia of Animals: 2020 Edition (Kollias et al., 2023) and the ethical standards of the Jaseng Animal Care and Use Committee (Approval No. JSR-2023-01-005-A). To promote animal welfare, nesting materials were supplied to reduce stress, and humane endpoints were determined in line with the core principles established by the International Council for Laboratory Animal Science (Bradfield et al., 2018). Epidural catheterization for extended injections was standardized, as previously reported (Hong et al., 2023b). A drug injection device (Instech Laboratories, Inc., Plymouth Meeting, PA, United States) was embedded at the spinal C2 level. Meanwhile, a modified catheter was navigated from the epidural space between the T10 and T11 levels to the L4 level. Post-implantation, an 80-kpa silicone block was inserted into the spinal canal between L4 and L5. The surgical procedures for inducing LSS at the L4 level have been detailed previously (Kim et al., 2021a). After suturing, the rats were administered cefazolin sodium intramuscularly for 3 days and acetaminophen orally for 3 days. From the day of the epidural catheter placement and LSS procedure, Sh2 was administered epidurally five times per week for 4 weeks, at either one or 2 mg/kg concentration. The experimental rats were categorized into four groups: Naive, LSS, Sh2-1, and Sh2-2 (*n* = 20 rats/group). Notably, naive rats were exempted from catheter and LSS procedures, whereas LSS rats underwent both. The Sh2-1 and Sh2-2 rats were exposed to 1 and 2 mg/kg Sh2, respectively, via the implanted epidural catheter following LSS induction.

### 2.8 Immunohistochemistry

Immunohistochemistry (IHC) was performed according to the procedures described in previous studies (Kim et al., 2017; Kim et al., 2022). The L3–L6 vertebra was excised and post-fixed in 4% PFA (Biosesang) for 24 h at 4°C. Subsequently, the sample was decalcified in a decalcification solution (BBC Biochemical, Mount Vernon, WA, United States) for 3 days and cryoprotected using 30% sucrose. After axially sectioning the samples to a thickness of 20 μm and drying for 24 h, they were incubated with anti-ED1, anti-iNOS, anti-NeuN, anti-TRPV1, anti-NF200, IB4, anti-CGRP, and anti-5HT primary antibodies overnight at 4°C. The next day, samples were rinsed and incubated with secondary antibodies: FITC-conjugated goat anti-mouse or rabbit IgG, rhodamine goat anti-rabbit or guinea pig IgG, or Alexa Fluor 647 goat anti-mouse IgG, for 2 h at room temperature. After further rinsing with PBS, samples were processed with DAKO mounting medium (Dako Cytomation, Glostrup, Denmark). Images were captured using a confocal microscope (Eclipse C2 Plus) at 10× or ×40 magnifications. Quantitative analysis of relative intensity was carried out using ImageJ software, with cells displaying particular markers counted manually. Initially, a rolling ball algorithm in ImageJ tools was used to subtract the background. We then measured the intensity or proportion of cells that showed brightness when exposed to certain antibodies (ED1, iNOS, TRPV1, IB4, CGRP, NF200, and 5-HT).

### 2.9 Real-time polymerase chain reaction

The RNeasy Mini Kit (Qiagen, Hilden, Germany) was used for isolating total RNA from the L4 level of the spinal cord. Oligo-dT-primed cDNAs were synthesized using the AccuPower RT PreMix (Bioneer, Daejeon, Korea). Real-time polymerase chain reaction (PCR) was executed with the CFX Connect Real-Time PCR Detection System (Bio-Rad, Hercules, CA, United States). The primer sequences are detailed in Table 1. The expression of target genes was normalized against that of glyceraldehyde 3-phosphate dehydrogenase encoding gene and is presented as a fold change difference.

**TABLE 1:** Primer sequences used for real-time polymerase chain reaction analysis.

Gene	5′–3′	Primer sequence
*INOS*	Forward	ATG​GCT​TGC​CCC​TGG​AAG​TT
	Reverse	TGT​TGG​GCT​GGG​AAT​AGC​AC
*IL-1β*	Forward	TTG​CTT​CCA​AGC​CCT​TGA​CT
	Reverse	GGT​CGT​CAT​CAT​CCC​ACG​AG
*TNF-α*	Forward	CCG​ACT​ACG​TGC​TCC​TCA​CC
	Reverse	CTC​CAA​AGT​AGA​CCT​GCC​CG
*COX2*	Forward	CTC​AGC​CAT​GCA​GCA​AAT​CC
	Reverse	GGG​TGG​GCT​TCA​GCA​GTA​AT
*IL-10*	Forward	CCT​GCT​CTT​ACT​GGC​TGG​AG
	Reverse	CCT​GGG​GCA​TCA​CTT​CTA​CC
*Arg1*	Forward	GTC​TCC​AGA​TGC​CTT​TGC​TTC
	Reverse	ATG​AAA​TTC​AGG​GTG​TGG​GAA​T
*SCN9a*	Forward	ACA​GTC​CCT​TGC​CCT​CAT​TG
	Reverse	AGT​ATG​GGT​CCA​GGT​CCT​CC
*IL1RN*	Forward	AAG​ACC​TTC​TAC​CTG​AGG​AAC​AAC​C
	Reverse	GCC​CAA​GAA​CAC​ATT​CCG​AAA​GTC
*TRPV1*	Forward	TTC​ACC​GAA​TGG​GCC​TAT​GG
	Reverse	TCA​CTG​CTG​CTG​TAA​GCG​AT
*NF200*	Forward	AAC​ACC​ACT​TAG​ATG​GCG​GG
	Reverse	ACG​TGG​AGC​GTT​CAG​CAA​TA
*GAPDH*	Forward	CCC​CCA​ATG​TAT​CCG​TTG​TG
	Reverse	TAG​CCC​AGG​ATG​CCC​TTT​AGT

### 2.10 Tissue clearing

A previously reported tissue-clearing method was utilized to generate transparent and high-definition images of DRG tissues (Renier et al., 2016; Zhu et al., 2021). Initially, DRG tissues were fixed using 4% PFA solution and subsequently washed with PBS. Decolorization was achieved using CUBIC-L solution containing 10% Triton X-100 (Sigma-Aldrich, United States) and 10% N-butyldiethanolamine (Tokyo Chemical Industry, Japan). A subsequent PBS wash removed residual CUBIC-L. A lipid-removal regimen entailed a stepwise dehydration using graded series of methanol solution (Duksan, Korea; 20%, 40%, 60%, 80%, and 100%), each lasting 1 h. For complete lipid extraction, samples were incubated at 4°C overnight in a mixture of 66% dichloromethane (DCM; Sigma-Aldrich, United States) and 33% methanol. For optimal clarity and immunostaining, samples were submerged in 5% H_2_O_2_ (Sigma-Aldrich, United States) solution overnight at 4°C. A subsequent rehydration process involved stepwise incubation in a series of graded methanol (80%, 60%, 40%, and 20%) followed by PBS washes, each lasting an hour. Optimal tissue penetration during immunostaining was ensured through a 2-day permeabilization using 20% dimethyl sulfoxide (DMSO; Sigma-Aldrich, United States), 0.3 M glycine (Sigma-Aldrich, United States), and 0.2% Triton X-100 (PTx2) in PBS at 37°C. Post-permeabilization, samples were blocked in 10% DMSO and 6% normal goat serum (NGS; Vector Laboratories Inc., Newark, CA, United States) blocking solution within PTx2 over 2 days at 37°C to minimize non-specific antibody binding. For antigen labeling, TRPV1 and NeuN primary antibodies were diluted to 1:100 and 1:500, respectively, in PTwH solution containing 5% DMSO, 3% NGS, 0.2% Tween 20 (Sigma-Aldrich, United States), and 0.1% heparin (Sigma-Aldrich, United States). This incubation lasted 3 weeks at 37°C. The frequency of the washes in the 4-day period ensured the removal of unbound antibodies. Samples were then exposed to FITC- or rhodamine-conjugated secondary antibodies, followed by a PTwH wash to eliminate excess antibodies. The samples were dehydrated with methanol before being immersed in a 66% DCM and 33% methanol mix for 3 h at room temperature. For refractive index alignment, samples were submerged in dibenzyl ether (DBE; Sigma-Aldrich, United States) for 15 min. Finally, images of TRPV1 and NeuN-stained, cleared DRG tissues were captured using a confocal microscope (Eclipse C2 Plus; Nikon).

### 2.11 Behavioral analysis

A combination of four unique tests was utilized to assess motor function post-LSS induction and subsequent epidural Sh2 injection. Footprint analysis was conducted following previously established methods (Vincelette et al., 2007; Lakes and Allen, 2016). Rats’ hind limbs were inked in black, and the animals were permitted to tread over a 50 × 13 cm white paper strip. This method was repeated three times, and the footprints were analyzed for several parameters. Gait evaluation entailed measurements of stride length, step length, and toe-out angle. Motor function was gauged using the Basso, Beattie, and Bresnahan (BBB) scale as previously described (Kim et al., 2022). Over a span of 4 min, the BBB test was conducted on a non-slip acrylic surface and evaluated by two independent observers. On the BBB scale, a score of 0 indicates “no observable hindlimb movement,” while a score of 21 represents normal hindlimb movement. To assess motor balance, the ladder walking test was implemented and has been extensively documented in previous studies (Davaa et al., 2021). Rats had to cross a metallic pathway with uniformly spaced grids on three occasions. Their activity was recorded with a digital camera. The ladder score was derived from the ratio of incorrect hind limb steps to the overall number of hind limb steps, to reflect functional performance. Locomotor function was assessed at weekly intervals for 1 month for all experimental groups.

To measure pain sensitivity, the von Frey test was applied, which recorded paw withdrawal latencies in response to central mechanical stimulation on both hind paws using a von Frey filament (Ugo Basile, Varese, Italy) (Ferreira-Gomes et al., 2008). The averages from three or more readings were taken for precision. During these assessments, a digital camera recorded the rats’ activities, and two observers who were blinded to the treatment conditions evaluated the results. Regarding the sequence of analyses, the BBB test was conducted first, followed by the Von Frey test and then the Ladder test.

### 2.12 Statistical analysis

Data are presented as mean ± standard deviation. Group comparisons were performed using a one-way analysis of variance followed by Tukey’s *post hoc* analysis (GraphPad Prism, GraphPad, Inc., La Jolla, CA, United States). *p*-values <0.05 were deemed statistically significant.

## 3 Results

### 3.1 Sh2 enhances recovery in H_2_O_2_-injured sensory axons of primary DRG neurons

**FIGURE 1:**
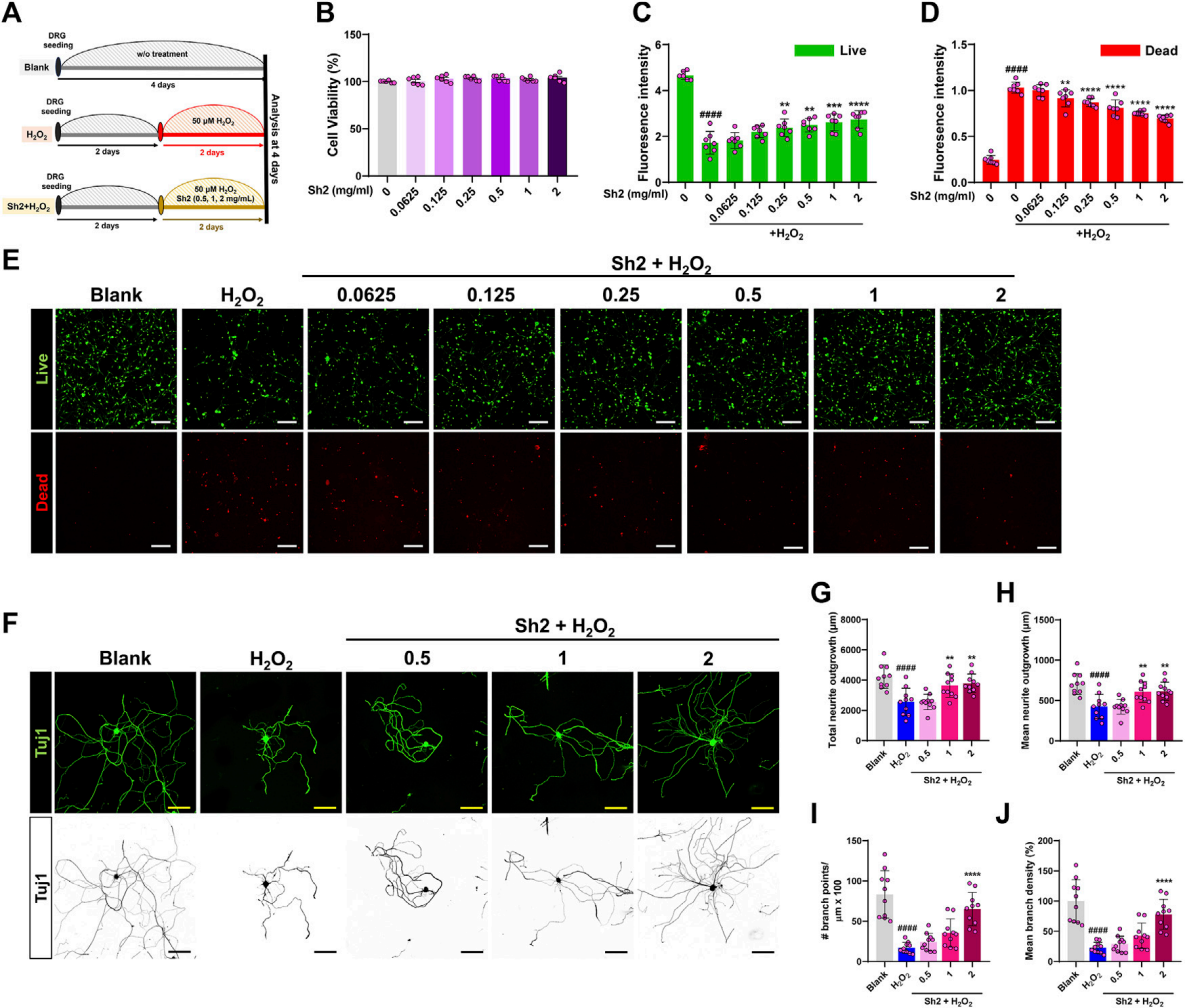
Sh2 enhances axonal growth and confers neuroprotection to primary cultured dorsal root ganglion (DRG) neurons in an H_2_O_2_ environment. **(A)** Schematic representation of the *in vitro* study timeline: 2 days of stabilization followed by 2 days of treatment using varying concentrations of Sh2 (0.5, 1, 2 mg/mL) in combination with H_2_O_2_; results were analyzed on day 4. **(B)** CCK assay detailing the response of primary cultured DRG neurons to a Sh2 concentration gradient (range: 0.0625–2 mg/mL) over 24 h, without H_2_O_2_ exposure. **(C and D)** Relative fluorescence intensities indicating cell viability; live cells marked green and dead cells marked red, demonstrating Sh2’s neuroprotective capacity against H_2_O_2_ damage. **(E)** Representative photomicrographs of cells, live cells in green, dead cells in red, under control, H_2_O_2_ alone, and combined Sh2 (range: 0.0625–2 mg/mL) and H_2_O_2_ conditions. White scale bar represents 200 μm. **(F)** Photomicrographs of representative DRG neurons labeled for Tuj1 (displayed in either green or black) post-treatment with control, H_2_O_2_ alone, or Sh2 (0.5, 1, 2 mg/mL) combined with H_2_O_2_. The yellow and black scale bars are set at 100 μm. **(G and H)** Quantitative assessment of total and average neurite lengths per neuron under control, H_2_O_2_ alone, or combined Sh2 (0.5, 1, or 2 mg/mL) and H_2_O_2_ conditions. **(I and J)** Metrics quantifying the count and average density of branch points for DRG neurites on a single-cell basis across all experimental groups. Data are presented as mean ± standard deviation. Significant variations were evaluated using one-way analysis of variance (ANOVA) coupled with Tukey’s *post hoc* analysis. Significance levels are denoted as follows: ^####^ *p* < 0.0001 compared with control group; ^**^ *p* < 0.01, ^***^ *p* < 0.001, and ^****^ *p* < 0.0001 compared with the H_2_O_2_ group.

The DRG houses a variety of sensory neuron subtypes essential for transmitting sensory data to the spinal cord and identifying painful environmental stimuli. We cultured primary DRG neurons in different combinations of Sh2 and H_2_O_2_ to ascertain Sh2’s ability to protect these neurons and promote sensory axon growth when exposed to H_2_O_2_ damage (Figure 1A). Upon treating DRG neurons with various concentrations of Sh2 (0.0625–2 mg/mL), we observed significant neuronal preservation without toxic repercussions (Figure 1B). Confirming our CCK-8 assay findings, the live/dead imaging assessment illuminated the neuroprotective capabilities of Sh2 under H_2_O_2_-induced stress. Specifically, H_2_O_2_-treated cells exhibited a noticeable decline in live cell (green) staining and an uptick in dead cell (red) staining. However, Sh2 intervention improved these metrics, showcasing more vibrant green and subdued red staining than the untreated control. These neuroprotective tendencies of Sh2 were dose-responsive, with observable changes beginning at 0.25 mg/mL Sh2 (green) and 0.0125 mg/mL (red) (Figures 1C–E). In sync with its neuroprotective traits, Sh2 application following H_2_O_2_ treatment bolstered the axonal growth of DRG neurons (Figure 1F). Detailed imaging of Tuj1-stained DRG neurons revealed more extended neurites in Sh2-treated neurons (1 and 2 mg/mL) than in those exposed to H_2_O_2_ alone (Figure 1G). This dose-responsive trend persisted when assessing mean neurite lengths between the 1 and 2 mg/mL Sh2 groups and the H_2_O_2_ group (Figure 1H). Sh2-treated DRG neurons also showed enhanced axonal branching. Notably, the branch points emanating from these neurites were significantly more abundant in the 2 mg/mL Sh2 group than in the H_2_O_2_ group (Figure 1I). Likewise, branch density measurements mirrored this trend, displaying clear distinctions between the 2 mg/mL Sh2 and H_2_O_2_ samples (Figure 1J).

### 3.2 Repeated epidural Sh2 injections diminish inflammation in LSS

**FIGURE 2:**
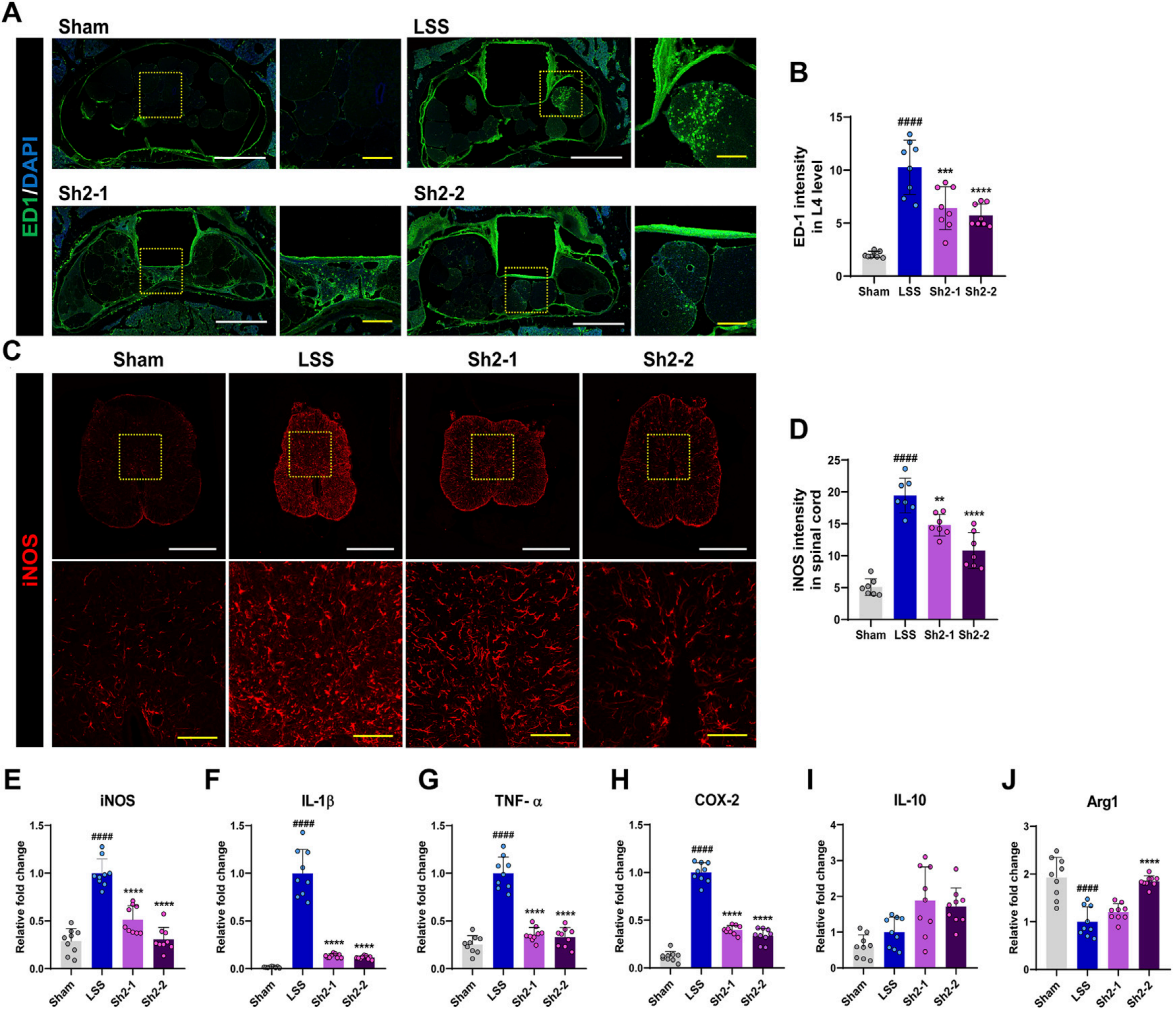
Epidural Sh2 administration modulates ED1^+^ macrophage activity and *iNOS* expression, attenuating inflammation. **(A)** Photomicrographs at 4 weeks, illustrating ED1^+^ signals (green) that mark activated macrophages upon inflammatory responses. White scale bar corresponds to 1 mm, yellow scale bar 20 μm. **(B)** Intensity quantification of ED1^+^ macrophages (green) at 4 weeks after lumbar spinal stenosis (LSS) induction, with and without periodic Sh2 epidural injections. **(C)** Photomicrographs highlighting variations in iNOS^+^ expression across spinal cord samples from different groups at 4 weeks. Scale bars: white = 1 mm, yellow = 20 μm. **(D)** Intensity quantification for iNOS^+^ cells across spinal cord samples from each experimental group. **(E–H)** Real-time PCR results delineating relative mRNA expression levels of pro-inflammatory markers, *iNOS*, *IL-1*β, *TNF-*α, and *COX-2*. **(I and J)** Real-time PCR data representing mRNA levels for anti-inflammatory markers, namely, *IL-10* and Arg*1*. All data are presented as mean ± standard deviation. Statistical distinctions were drawn using one-way analysis of variance (ANOVA) complemented by Tukey’s *post hoc* test. Significance demarcations are as follows: ^####^ *p* < 0.0001 compared with naive group; ^**^ *p* < 0.01, ^***^ *p* < 0.001, and ^****^ *p* < 0.0001, compared with lumbar spinal stenosis (LSS) group.

We aimed to understand the impact of long-term Sh2 administration via the epidural route after inducing LSS. We focused on the L4 level, precisely where a silicone block was implanted, and detected the presence of ED1^+^ macrophages and vital inflammation indicators after 4 weeks (Figure 2A). While these macrophages were clustered in small groups around the silicone insert, their prevalence was notably curtailed in the Sh2 epidural group than were in the LSS cohort. Quantitative analyses revealed a pronounced reduction in ED1^+^ macrophage intensity when Sh2 was delivered epidurally, underlining Sh2’s potential in countering LSS-induced inflammation. Notably, different concentrations of epidurally administered Sh2 yielded similar outcomes, lacking discernible variance (Figure 2B). Furthermore, we investigated changes in iNOS expression, a renowned inflammation instigator, in the aftermath of consistent epidural Sh2 delivery at LSS-impacted spinal cord regions. A surge in iNOS expression was evident throughout the 4 weeks post-LSS induction. Contrarily, the Sh2-treated group witnessed remarkable iNOS decline, even when matched against consistent capture gains (Figure 2C). Numerical evaluations affirmed these findings, highlighting a marked reduction in iNOS intensity among the Sh2 recipients vis-à-vis the LSS group, leading to dose-controlled mitigation (Figure 2D).

Finally, we evaluated the expression patterns of critical pro-inflammatory genes (*iNOS, IL-1*β*, TNF-*α*, COX-2*) following serial epidural Sh2 injections in LSS-stricken rats. Post-LSS, a sharp increase was apparent in the expression levels of these genes. However, consistent Sh2 injections displayed a dampening effect on these levels (Figures 2E–H). While evaluating the expression of IL-10, a potent anti-inflammatory cytokine, we observed an inclining trend in the Sh2 cohort, even if statistically insignificant. Interestingly, expression of another anti-inflammatory mediator, Arg1, was suppressed post-LSS. However, a 2 mg/kg Sh2 dosage led to a significant expression surge of Arg*1*, emphasizing its potential anti-inflammatory role (Figures 2I,J).

### 3.3 TRPV1 expression reduced in the lumbar DRG following repeated epidural Sh2 injections in LSS

**FIGURE 3:**
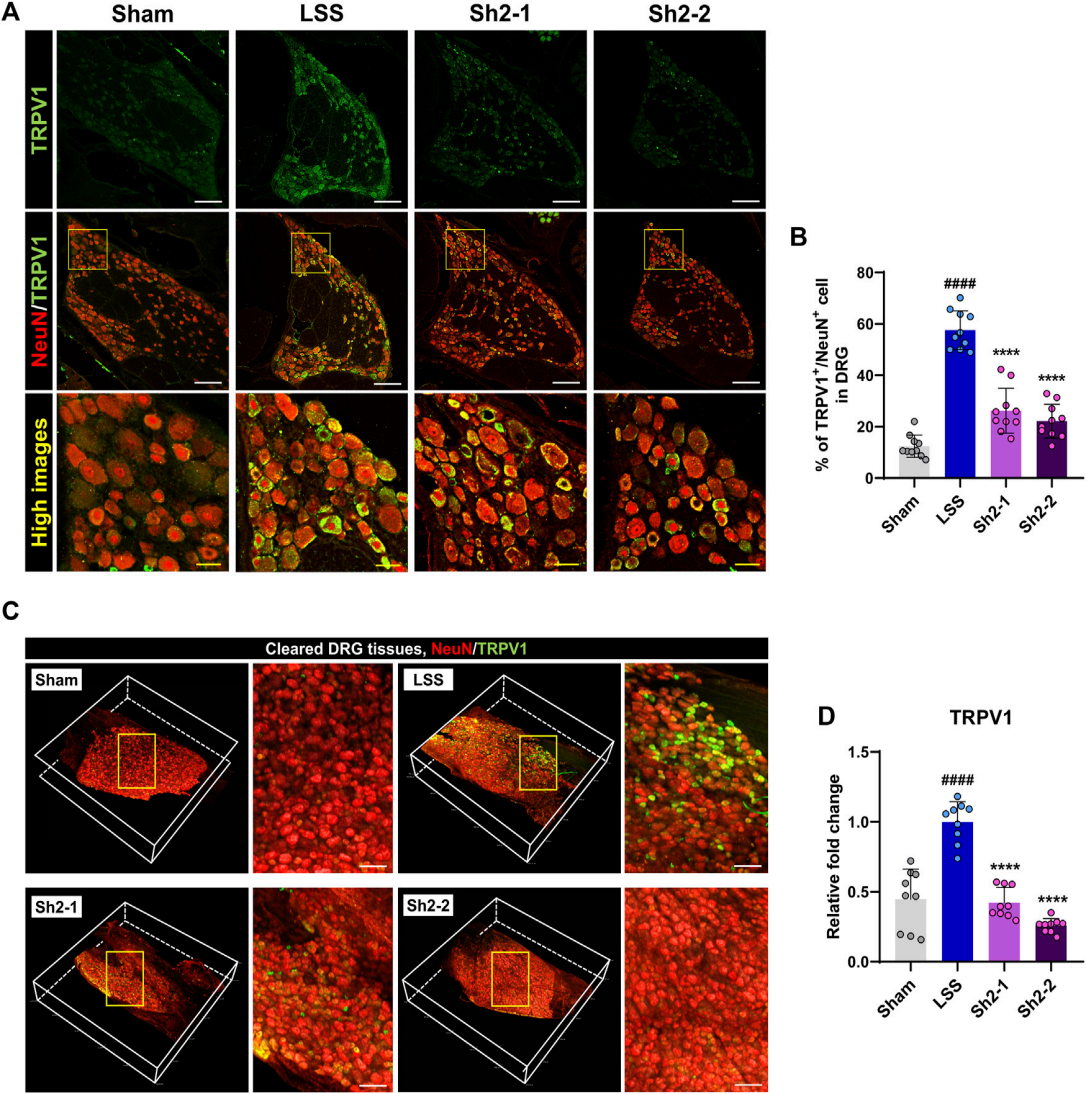
Epidural Sh2 administration mitigates TRPV1-mediated pain in lumbar spinal stenosis (LSS)-afflicted rats. **(A)** Photomicrographs at the 4-week mark depict TRPV1^+^ neurons (green) and NeuN^+^ neurons (red) in dorsal root ganglion (DRG) tissues across the studied groups. White scale bar, 200 μm; yellow bar, 20 μm. **(B)** Mean percentage of TRPV1^+^ neurons co-labeled with NeuN^+^ in DRG tissues from different groups. **(C)** 3D visualizations of cleared DRG samples stained for TRPV1 (green) and NeuN (red) from each study group. White scale bar, 500 μm. **(D)** Real-time PCR results detailing relative mRNA expression levels of *TRPV1* linked with inflammatory pain attenuation. Data are presented as mean ± standard deviation. Statistical differences were determined using one-way analysis of variance followed by Tukey’s *post hoc* analysis: ^####^ *p* < 0.0001 compared with naive group; ^****^ *p* < 0.0001 compared with LSS group.

TRPV1 receptors, which are the key facilitators in the transmission of inflammatory pain, can be inhibited to reduce such pain (Sondermann et al., 2019). Four weeks post-LSS induction, immunohistochemical examinations revealed a noticeable upregulation in TRPV1 expression within DRG tissue. However, the Sh2 group exhibited a marked decrease in TRPV1 expression (Figure 3A). Quantitative analysis demonstrated a significant reduction in the proportion of TRPV1^+^ neurons among NeuN-stained neurons in DRG tissue following repeated epidural Sh2 injections. This effect was notably prominent in both Sh2 groups compared to the LSS group. Importantly, the reduction was not dose-dependent (Figure 3B). To provide a more comprehensive view, we conducted tissue clearing and volumetric imaging of both TRPV1 and NeuN within the DRG tissue (Supplementary Video S1). A robust presence of TRPV1^+^ sensory cells was detected throughout the DRG tissue after LSS induction. However, animals subjected to epidural Sh2 injections post-LSS exhibited a significant decline in TRPV1^+^ neurons (Figure 3C). In line with these observations, *TRPV1* gene expression was sharply elevated post-LSS but saw a substantial and dose-dependent reduction following epidural Sh2 treatments, bolstering Sh2’s potential as a TRPV1 modulator in reducing inflammatory pain (Figure 3D).

### 3.4 Pain-associated neuronal subsets are modulated in the lumbar DRGs by repeated epidural Sh2 injections following LSS

**FIGURE 4:**
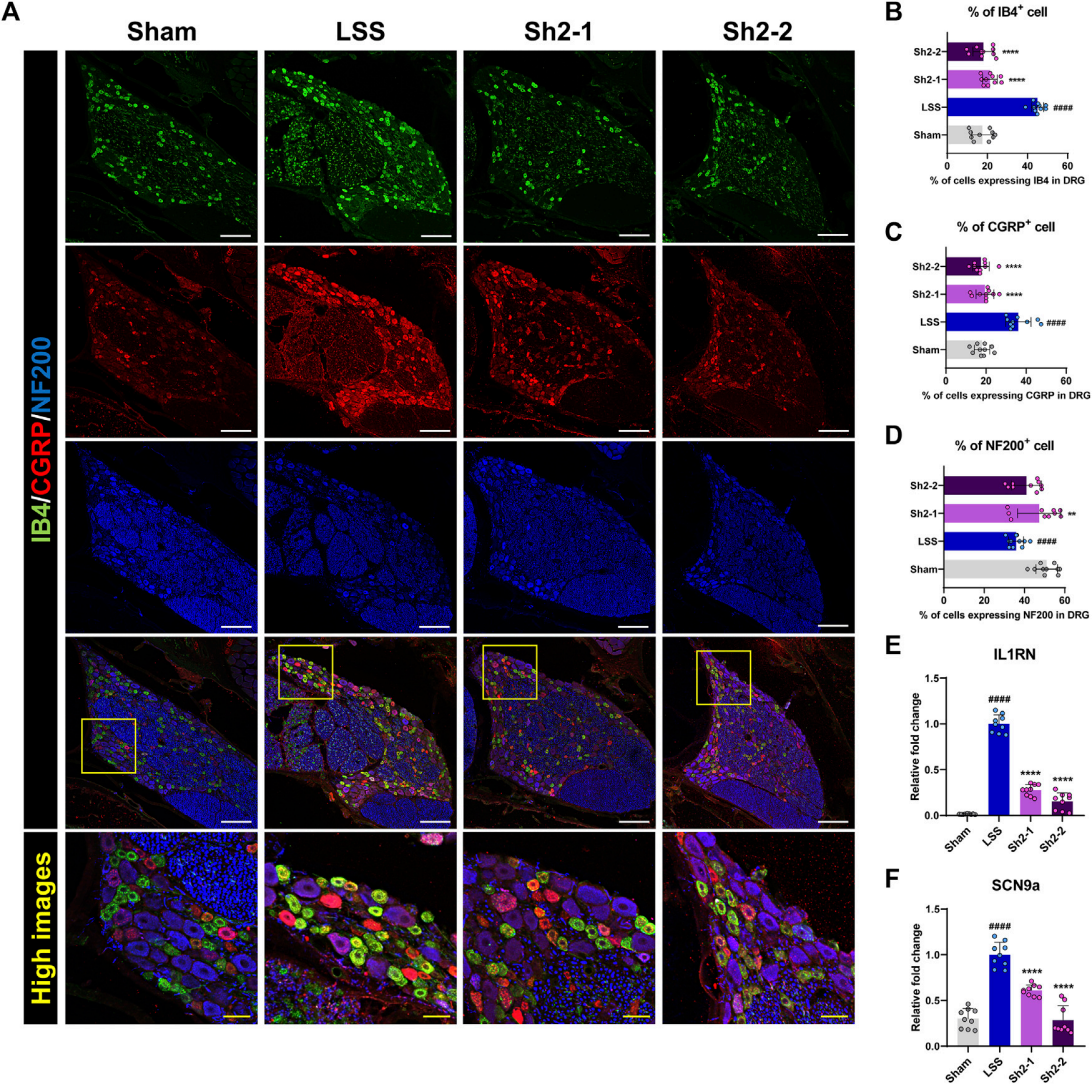
Epidural Sh2 administration modulates sensory neuron subtypes involved in pain regulation in lumbar spinal stenosis (LSS) rats. **(A)** Photomicrographs at 4 weeks presenting IB4^+^ (green), CGRP^+^ (red), and NF200^+^ (blue) neurons in dorsal root ganglion (DRG) tissues across groups. White scale bar, 200 μm; yellow scale bar, 50 μm. **(B–D)** Differential counts of neuronal subtypes (IB4, CGRP, and NF200) in DRG samples from each study group. **(E and F)** Real-time PCR results of relative mRNA expression levels for *IL1RN* and *SCN9a*, both integral to pain modulation. Data are presented as mean ± standard deviation. Significant deviations were determined through one-way analysis of variance, complemented by Tukey’s *post hoc* analysis: ^####^ *p* < 0.0001 compared with naive group; ^**^ *p* < 0.01 and ^****^ *p* < 0.0001 compared with LSS group.

To further delineate the specific DRG neurons contributing to the analgesic effects of Sh2, we performed immunostaining using three distinct neuronal markers—IB4, CGRP, and NF200—that are considered key indicators for identifying subsets of small-sized neurons implicated in pain sensation. Specifically, IB4 expression identifies neurons without peptide content involved in pain transmission after nerve injuries, while NF200 serves as a marker for both small and large myelinated fibers associated with pain and proprioception (Kuniyoshi et al., 2007). This multifaceted approach allowed for a comprehensive evaluation of DRG neuronal populations influenced by Sh2 (Figure 4A).

Post-LSS induction, a marked increase in the number of neurons expressing IB4 or CGRP was observed. Conversely, administering repeated epidural Sh2 injections led to a significant decrease in the number of these neurons. Interestingly, the proportions of IB4^+^ and CGRP^+^ neurons did not vary significantly across Sh2 concentrations (Figures 4B,C). Additionally, NF200^+^ neurons, which were significantly reduced after LSS induction, were notably increased in rats treated with 1 mg/kg Sh2 (Figure 4D). Furthermore, we observed significantly upregulated expression of pain-related genes, such as *IL1RN* and sodium voltage-gated channel alpha subunit 9 (*SCN9a*), in the LSS group compared to the control group. Epidural Sh2 administration significantly dampened the expression of these genes in a dose-dependent manner (Figures 4E,F). This further underscores the potential of Sh2 in modulating various subsets of neurons involved in pain perception and transmission, thereby serving as a versatile therapeutic agent for alleviating LSS-induced discomfort.

### 3.5 Axonal sprouting stimulation and enhanced functional recovery in LSS via repeated epidural Sh2 injections

**FIGURE 5:**
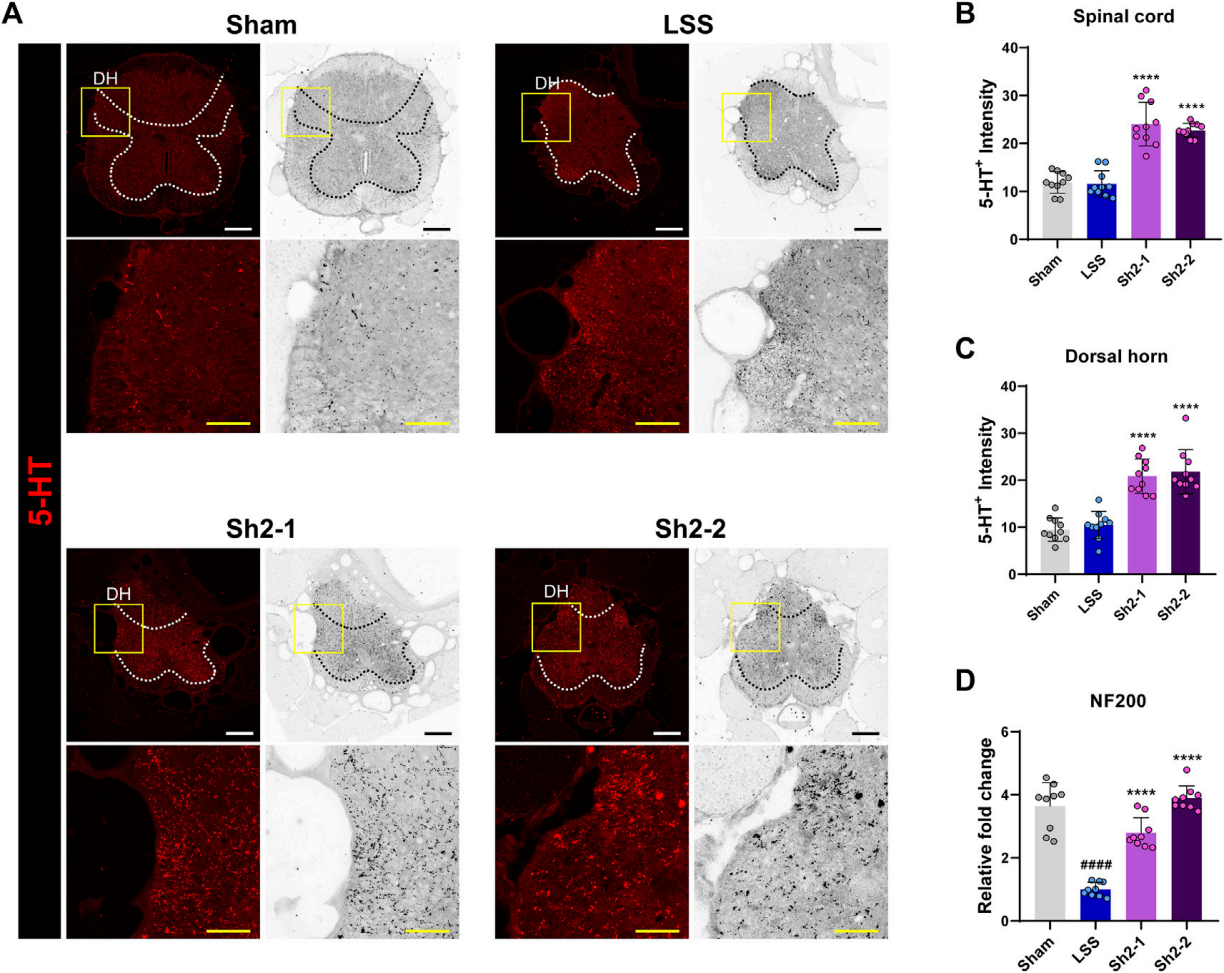
Epidural Sh2 administration fosters 5-HT^+^ axonal growth, particularly in the spinal cord’s dorsal horn. **(A)** Photomicrographs at 4 weeks illustrating 5-HT^+^ (red) axons in the spinal cord across groups. White and black scale bars, 200 μm; yellow scale bar, 40 μm. **(B and C)** Intensity analysis of 5-HT^+^ axons throughout the spinal cord and more specifically in the dorsal horn region for each study group. **(D)** Real-time PCR data indicating relative mRNA expression levels of *NF200*, linked to axonal regeneration. Data are presented as mean ± standard deviation. Significant differences were discerned using one-way analysis of variance, augmented by Tukey’s *post hoc* analysis: ^####^ *p* < 0.0001 compared with naive group; ^****^ *p* < 0.0001 compared with LSS group.

The role of axonal sprouting of serotonin fibers in kickstarting locomotor activity is well-acknowledged (Ghosh and Pearse, 2014). We thus sought to determine if repeated epidural injections of Sh2 could enhance the sprouting of 5HT^+^ axons within the L4 spinal cord region. Impressively, a heightened presence of 5HT^+^ axons was observed in the spinal cord of those in the Sh2 group (Figure 5A). Quantitative analysis showed that the Sh2 group displayed a markedly stronger 5HT^+^ signal throughout the spinal cord when compared to the LSS group (Figure 5B). This discrepancy was especially pronounced within the dorsal horn region of the spinal cord (Figure 5C), although variations in concentrations among the Sh2 group did not yield significant differences. To further evaluate the potential regenerative capacity of axons following compression damage, we conducted NF200 staining. Unlike the distinct variations observed with 5HT^+^ axon sprouting, the groups showed negligible differential responses in NF200 axonal immune signals (Supplementary Figure S1). However, while the NF200 axonal effects were not readily visible, the *NF200* mRNA expression demonstrated a noticeable dose-dependent elevation in the Sh2 group at 4 weeks (Figure 5D). Collectively, these observations strongly suggest that repeated epidural Sh2 injections can augment the growth of 5HT^+^ axons post-LSS, which, in turn, may be instrumental in aiding functional recovery.

**FIGURE 6:**
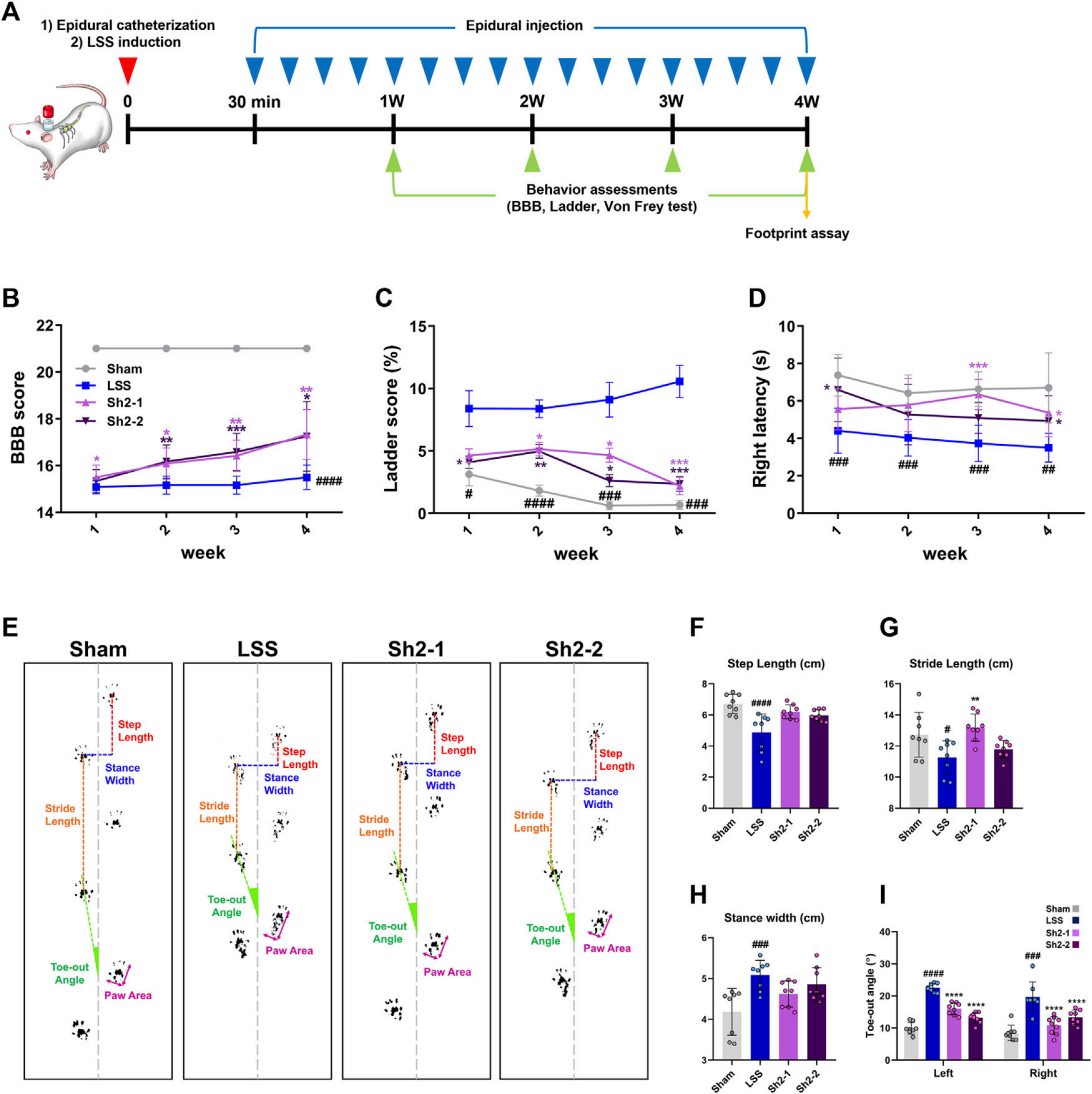
Repeated delivery of Sh2 through the epidural space improves locomotor functions over 4 weeks in lumbar spinal stenosis (LSS) rats. **(A)** Schematic of the *in vivo* experiment schedule with repeated epidural Sh2 injection five times per week and weekly behavioral testing for 4 weeks **(B)** Basso, Beattie, and Bresnahan (BBB) scale score using open field test, **(C)** missed step % on ladder rung walking test, and **(D)** right latency on von Frey test. **(E)** Representative images showing footprint analysis for each group at 4 weeks **(F–I)** Quantitative analysis of footprint records, including step length (cm), stride length (cm), stance width (cm), and toe-out angle (º) in each group. Data are expressed as the mean ± standard deviation. Significant differences were analyzed via one-way analysis of variance with Tukey’s post-hoc analysis as follows: ^##^ *p* < 0.01, ^###^ *p* < 0.001, and ^####^ *p* < 0.0001 compared with naive group; ^*^ *p* < 0.05, ^**^ *p* < 0.01, ^***^ *p* < 0.001, and ^****^ *p* < 0.0001 compared with LSS group.

To gauge the functional ramifications of these findings, we monitored animals’ locomotor capabilities weekly for a period of 4 weeks using the BBB scale alongside ladder scoring according to the schedule shown in Figure 6A. A clear pattern emerged of the Sh2-treated group rats having notably superior BBB locomotor recovery than the LSS group. The rats in the group administered 1 mg/kg Sh2 displayed significant improvements from the first week, while those in the 2 mg/kg Sh2 group manifested improvements from the second week onward, with these differences becoming progressively pronounced over the full 4-week duration (Figure 6B). The ladder scoring assessments corroborated the promising therapeutic potential of Sh2, as the treated group displayed fewer ladder missteps than the LSS group. These differences became apparent from the first week in the 2 mg/kg Sh2 group and from the second week in the 1 mg/kg Sh2 group, with consistent improvements observed throughout the 4-week study (Figure 6C). Furthermore, we employed the von Frey test to examine mechanical sensitivity on a weekly basis for the 4-week period. Rats subjected to repeated epidural Sh2 injections exhibited considerably longer latency period in their right hind limb as opposed to LSS rats. Specifically, the 1 mg/kg Sh2 group demonstrated a significant difference at the 3-week point, while the 2 mg/kg Sh2 group showed marked differences during the first and fourth weeks. Even though these variations were not consistently observed across the 4 weeks, the average values in the Sh2 group closely mirrored the values of the naive group, as opposed to the values of the LSS group (Figure 6D). Finally, the gait characteristics of the hind limbs were assessed using footprint analysis at the 4-week endpoint (Figure 6E). When evaluating gait characteristics—stride length, step length, and toe-out angle—we based our analysis on three successive hind limb footprints. LSS-affected rats presented a discernibly altered hind limb gait, compared to the naive rats. Specifically, stride and step lengths were markedly reduced in LSS rats, accompanied by a noticeable outward turning of the feet. Conversely, rats subjected to repeated epidural Sh2 injections displayed mean stride and step lengths resembling those of the naive rats, suggesting a reversion towards normal gait patterns. However, these measurements were not statistically distinct from the LSS group (Figures 6F,G). In terms of stance width, the LSS group displayed a significantly wider stance compared to the naive group. While the Sh2-treated group demonstrated a tendency toward a narrower stance width, the differences across the groups were not statistically significant (Figure 6H). A striking feature post-LSS was a pronounced increase in the toe-out angle for both left and right hind limbs. Remarkably, recurrent epidural Sh2 injections in LSS rats facilitated a return to nearly trunk-parallel toe angles, showcasing a dose-dependent response in the case of the left hind limb when juxtaposed with the naive group (Figure 6I). Notably, the elevated BBB scores observed in the Sh2 treatment group had significant implications, suggesting that repeated epidural Sh2 injections hold the potential to enhance motor function.

## 4 Discussion

This study explored the therapeutic potential of repeated epidural Sh2 injections for managing inflammation, altering pain-associated neuronal clusters, promoting axonal sprouting, and enhancing functional recovery following LSS. Historically, Sh2 pharmacopuncture has been intramuscularly administered directly to the acupoint at the injury site, with its efficacy being corroborated through both clinical and preclinical studies (Lee et al., 2017; Park et al., 2019; Kim et al., 2021b; Kim et al., 2023). We explored the epidural route for Sh2 administration, since enhancing its potency was the primary objective. Distinct from prior research, which generally centered on a single epidural delivery (Suto et al., 2012; Nahm et al., 2017; Lee et al., 2021), our approach pioneered a structured protocol for consistent epidural injections, considerably extending the duration of Sh2 epidural administration (Hong et al., 2023b). Given that continuous epidural nerve block is primarily used for pain mitigation (Dong et al., 2021), primary cultured DRG neurons were selected for *in vitro* examinations, providing insights into Sh2’s influence on neuroprotective modulation. The evident regeneration of sensory axons in primary cultured DRG neurons, especially after H_2_O_2_-induced damage, highlights the potential neuroprotective nature of Sh2. Immunostaining was evaluated using pain-related markers (TRPV1, IB4, and CGRP), but no differences were noted between the blank and H_2_O_2_ groups. Our research focuses on exploring the potential of Sh2 injections as an alternative to traditional steroid injections, aiming to improve the treatment methods for spinal stenosis clinically (Manchikanti et al., 2015). To achieve this, we employed a LSS animal model that accurately reflects the clinical conditions of human spinal stenosis. This model was carefully selected and developed according to established methods that effectively induce LSS (Kim et al., 2021a). For extradural applications of Sh2, we selected concentrations of 1 and 2 mg/kg for repeated epidural injections in the LSS model. These concentrations mirror those of 1 and 2 mg/mL, which were proven to be safe and effective in *vitro* experiments. Since epidural injections are administered close to neural tissues, they can directly impact nerve root (Eckel and Bartynski, 2009), potentially replicating the effects of direct drug administration to nerve cells observed *in vitro*. However, *in vitro* experiments cannot fully capture the complexities of the *in vivo* environment, where the processes of drug absorption, distribution, metabolism, and excretion significantly influence the drug’s ultimate impact. Consequently, additional research was conducted in the LSS model with these concentrations to achieve comparable results *in vivo.* Repeated epidural injections of Sh2 in LSS rats led to a marked decline in ED1^+^ macrophages and iNOS expression, illustrating a modulation of the inflammatory pathways that might intensify LSS-related neurological symptoms. Enhanced Arg1 expression and a diminished pro-inflammatory gene profile in the Sh2-administered groups accentuate the anti-inflammatory property of Sh2. Considering that TRPV1 inhibition typically results in subdued inflammatory pain, the decline in TRPV1 expression post-Sh2 epidural injections signified its prospective utility in pain modulation. Moreover, varied markers associated with DRG neurons furnished detailed insights into the specific DRG neuronal subsets impacted by Sh2. For instance, modulation of IB4, CGRP, and NF200^+^ neurons suggests that Sh2 may affect different pain pathways. Evaluations of pain-related genes such as *IL1RN* and *SCN9a* offered a deeper understanding of the molecular mechanisms underpinning these neural alterations. Notably, these genes are linked to pain perception and lumbar radicular discomfort (Bjorland et al., 2016; Zorina-Lichtenwalter et al., 2018). The substantial downregulation of these genes, evident in a dose-responsive manner upon Sh2 introduction to LSS rats, propounds the likely modus operandi of Sh2 in modulating the expression of pain-related genes, thereby enhancing its analgesic effects.

Furthermore, the increased presence of 5HT^+^ axons within the spinal cord, combined with the significant advancements in locomotor recuperation as discerned from BBB and ladder evaluations (Fouad et al., 2010; Ghosh and Pearse, 2014), demonstrates Sh2’s potential for fostering axonal growth and functional improvements. Collectively, these findings underscore the potential of Sh2 epidural injections as an effective treatment option for lumbar spinal stenosis (LSS), emphasizing their benefits in both alleviating symptoms and facilitating functional recovery. These discoveries support the consideration of Sh2 as a viable epidural therapeutic alternative for patients with LSS. Nevertheless, it is pivotal to underscore that the current research has some limitations, particularly concerning evaluations of the safety of Sh2 epidural applications. Comprehensive assessments, encompassing factors such as body weight, food intake, organ metrics, urinalysis, hematology, blood chemistry, and histopathology, are imperative for rats exposed to prolonged, repeated epidural Sh2 injections. Additionally, it remains unresolved whether Sh2, when delivered epidurally, surpasses its efficacy compared to other traditional administration routes. Thus, an in-depth comparative study, solely focusing on modifying the application pathways under unchanged conditions and dosages, is a forthcoming necessity. To further unravel the potential of Sh2, investigations should be steered toward ascertaining the safety associated with its epidural delivery, discerning potential adversities or interactions, determining if its most potent impact is through epidural administration by juxtaposing with other routes, and eventually, transitioning to clinical trials for human-centric validation of these outcomes.

## 5 Conclusion

In summary, this study provides compelling evidence that prolonged, repeated epidural injections of Sh2 are effective in promoting axonal sprouting, reducing inflammation, managing pain, and enhancing functional recovery in rats with lumbar spinal stenosis (LSS). The comprehensive analysis demonstrates Sh2’s therapeutic potential, setting the stage for further research into its optimal application in clinical settings, particularly through epidural administration.

## Data Availability

The original contributions presented in the study are included in the article/Supplementary Materials, further inquiries can be directed to the corresponding author.

## References

[B1] Bellini M. Barbieri M. (2013). Systemic effects of epidural steroid injections. Anaesthesiol. Intensive Ther. 45, 93–98. 10.5603/AIT.2013.0021 23877903

[B2] Bjorland S. Moen A. Schistad E. Gjerstad J. Roe C. (2016). Genes associated with persistent lumbar radicular pain; a systematic review. BMC Musculoskelet. Disord. 17, 500. 10.1186/s12891-016-1356-5 27964712 PMC5154161

[B3] Bradfield J. F. GuillAn J. Anderson L. C. (2018). “Harmonizing international animal care and use programs,” in Management of animal care and use programs in research, education, and testing. Editors Weichbrod R. H. Thompson G. A. Norton J. N. (Boca Raton (FL): Global Principles of Research Animal Welfare), 147–158.

[B4] Carassiti M. Pascarella G. Strumia A. Russo F. Papalia G. F. Cataldo R. (2021). Epidural steroid injections for low back pain: a narrative review. Int. J. Environ. Res. Public Health 19, 231. 10.3390/ijerph19010231 35010492 PMC8744824

[B5] Cohen S. P. Bicket M. C. Jamison D. Wilkinson I. Rathmell J. P. (2013). Epidural steroids: a comprehensive, evidence-based review. Reg. Anesth. Pain Med. 38, 175–200. 10.1097/AAP.0b013e31828ea086 23598728

[B6] Davaa G. Hong J. Y. Kim T. U. Lee S. J. Kim S. Y. Hong K. (2021). Exercise ameliorates spinal cord injury by changing DNA methylation. Cells 10, 143. 10.3390/cells10010143 33445717 PMC7828206

[B7] Dong X. Liu Y. Yang Q. Liu Z. Zhang Z. (2021). Comparison of therapeutic effects of continuous epidural nerve block combined with drugs on postherpetic neuralgia. Int. J. Neurosci. 131, 191–195. 10.1080/00207454.2020.1736583 32125200

[B8] Eckel T. S. Bartynski W. S. (2009). Epidural steroid injections and selective nerve root blocks. Tech. Vasc. Interv. Radiol. 12, 11–21. 10.1053/j.tvir.2009.06.004 19769903

[B9] Ferreira-Gomes J. Adaes S. Castro-Lopes J. M. (2008). Assessment of movement-evoked pain in osteoarthritis by the knee-bend and CatWalk tests: a clinically relevant study. J. Pain 9, 945–954. 10.1016/j.jpain.2008.05.012 18650131

[B10] Fornari M. Robertson S. C. Pereira P. Zileli M. Anania C. D. Ferreira A. (2020). Conservative treatment and percutaneous pain relief techniques in patients with lumbar spinal stenosis: WFNS spine committee recommendations. World Neurosurg. X 7, 100079. 10.1016/j.wnsx.2020.100079 32613192 PMC7322792

[B11] Fouad K. Rank M. M. Vavrek R. Murray K. C. Sanelli L. Bennett D. J. (2010). Locomotion after spinal cord injury depends on constitutive activity in serotonin receptors. J. Neurophysiol. 104, 2975–2984. 10.1152/jn.00499.2010 20861436 PMC3007654

[B12] Genevay S. Atlas S. J. (2010). Lumbar spinal stenosis. Best. Pract. Res. Clin. Rheumatol. 24, 253–265. 10.1016/j.berh.2009.11.001 20227646 PMC2841052

[B13] Ghai B. Vadaje K. S. Wig J. Dhillon M. S. (2013). Lateral parasagittal versus midline interlaminar lumbar epidural steroid injection for management of low back pain with lumbosacral radicular pain: a double-blind, randomized study. Anesth. Analg. 117, 219–227. 10.1213/ANE.0b013e3182910a15 23632053

[B14] Ghosh M. Pearse D. D. (2014). The role of the serotonergic system in locomotor recovery after spinal cord injury. Front. Neural Circuits 8, 151. 10.3389/fncir.2014.00151 25709569 PMC4321350

[B15] Gunzburg R. Szpalski M. (2003). The conservative surgical treatment of lumbar spinal stenosis in the elderly. Eur. Spine J. 12 (Suppl. 2), S176–S180. 10.1007/s00586-003-0611-2 12961080 PMC3591835

[B16] Ha C. W. Park Y. B. Kyung H. S. Han C. S. Bae K. C. Lim H. C. (2016). Gastrointestinal safety and efficacy of long-term GCSB-5 use in patients with osteoarthritis: a 24-week, multicenter study. J. Ethnopharmacol. 189, 310–318. 10.1016/j.jep.2016.05.031 27196293

[B17] Hong J. Y. Kim H. Lee J. Jeon W. J. Lee Y. J. Ha I. H. (2022). Harpagophytum procumbens inhibits iron overload-induced oxidative stress through activation of Nrf2 signaling in a rat model of lumbar spinal stenosis. Oxid. Med. Cell Longev. 2022, 1–18. 10.1155/2022/3472443 PMC9492433 36160714

[B18] Hong J. Y. Kim H. Lee J. Jeon W. J. Yeo C. Kim H. (2023b). Epidural injection method for long-term pain management in rats with spinal stenosis. Biomedicines 11, 1390. 10.3390/biomedicines11051390 37239061 PMC10216675

[B19] Hong J. Y. Lee J. Kim H. Yeo C. Jeon W. J. Lee Y. J. (2023a). Shinbaro2 enhances axonal extension beyond the glial scar for functional recovery in rats with contusive spinal cord injury. Biomed. Pharmacother. 168, 115710. 10.1016/j.biopha.2023.115710 37862963

[B20] Kim H. Hong J. Y. Jeon W. J. Lee J. Baek S. H. Ha I. H. (2021b). Lycopus lucidus turcz exerts neuroprotective effects against H(2)O(2)-induced neuroinflammation by inhibiting NLRP3 inflammasome activation in cortical neurons. J. Inflamm. Res. 14, 1759–1773. 10.2147/JIR.S305031 33981154 PMC8109151

[B21] Kim H. Hong J. Y. Jeon W. J. Lee J. Ha I. H. (2021a). Evaluation of the effects of differences in silicone hardness on rat model of lumbar spinal stenosis. PLoS One 16, e0251464. 10.1371/journal.pone.0251464 33984013 PMC8118556

[B22] Kim H. Hong J. Y. Jeon W. J. Lee J. Lee Y. J. Ha I. H. (2022). Melittin regulates iron homeostasis and mediates macrophage polarization in rats with lumbar spinal stenosis. Biomed. Pharmacother. 156, 113776. 10.1016/j.biopha.2022.113776 36244265

[B23] Kim J. K. Park S. W. Kang J. W. Kim Y. J. Lee S. Y. Shin J. (2012). Effect of GCSB-5, a herbal formulation, on monosodium iodoacetate-induced osteoarthritis in rats. Evid. Based Complement. Altern. Med. 2012, 730907. 10.1155/2012/730907 PMC3303749 22474519

[B24] Kim J. W. Mahapatra C. Hong J. Y. Kim M. S. Leong K. W. Kim H. W. (2017). Functional recovery of contused spinal cord in rat with the injection of optimal-dosed cerium oxide nanoparticles. Adv. Sci. (Weinh) 4, 1700034. 10.1002/advs.201700034 29051850 PMC5644223

[B25] Kim M. S. El-Fiqi A. Kim J. W. Ahn H. S. Kim H. Son Y. J. (2016). Nanotherapeutics of PTEN inhibitor with mesoporous silica nanocarrier effective for axonal outgrowth of adult neurons. ACS Appl. Mater Interfaces 8, 18741–18753. 10.1021/acsami.6b06889 27386893

[B26] Kim T. H. Yoon S. J. Lee W. C. Kim J. K. Shin J. Lee S. (2011). Protective effect of GCSB-5, an herbal preparation, against peripheral nerve injury in rats. J. Ethnopharmacol. 136, 297–304. 10.1016/j.jep.2011.04.037 21569830

[B27] Kim W. K. Shin J. S. Lee J. Koh W. Ha I. H. Park H. J. (2023). Effects of the administration of Shinbaro 2 in a rat lumbar disk herniation model. Front. Neurol. 14, 1044724. 10.3389/fneur.2023.1044724 36970511 PMC10036394

[B28] Kollias N. S. Hess W. J. Johnson C. L. Murphy M. Golab G. (2023). A literature review on current practices, knowledge, and viewpoints on pentobarbital euthanasia performed by veterinarians and animal remains disposal in the United States. J. Am. Vet. Med. Assoc. 261, 733–738. 10.2460/javma.22.08.0373 36800298

[B29] Kuniyoshi K. Ohtori S. Ochiai N. Murata R. Matsudo T. Yamada T. (2007). Characteristics of sensory DRG neurons innervating the wrist joint in rats. Eur. J. Pain 11, 323–328. 10.1016/j.ejpain.2006.05.003 16807014

[B30] Lakes E. H. Allen K. D. (2016). Gait analysis methods for rodent models of arthritic disorders: reviews and recommendations. Osteoarthr. Cartil. 24, 1837–1849. 10.1016/j.joca.2016.03.008 PMC5026889 26995111

[B31] Lee B. H. Moon S. H. Suk K. S. Kim H. S. Yang J. H. Lee H. M. (2020). Lumbar spinal stenosis: pathophysiology and treatment principle: a narrative review. Asian Spine J. 14, 682–693. 10.31616/asj.2020.0472 33108834 PMC7595829

[B32] Lee H. J. Ju J. Choi E. Nahm F. S. Choe G. Y. Lee P. B. (2021). Effect of epidural polydeoxyribonucleotide in a rat model of lumbar foraminal stenosis. Korean J. Pain 34, 394–404. 10.3344/kjp.2021.34.4.394 34593657 PMC8494961

[B33] Lee Y. J. Shin J. S. Lee J. Kim M. R. Ahn Y. J. Shin Y. S. (2017). Survey of integrative lumbar spinal stenosis treatment in Korean medicine doctors: preliminary data for clinical practice guidelines. BMC Complement. Altern. Med. 17, 425. 10.1186/s12906-017-1942-6 28851418 PMC5574237

[B34] Liu K. Liu P. Liu R. Wu X. Cai M. (2015). Steroid for epidural injection in spinal stenosis: a systematic review and meta-analysis. Drug Des. Devel Ther. 9, 707–716. 10.2147/DDDT.S78070 PMC4322611 25678775

[B35] Lurie J. Tomkins-Lane C. (2016). Management of lumbar spinal stenosis. BMJ 352, h6234. 10.1136/bmj.h6234 26727925 PMC6887476

[B36] Manchikanti L. Kaye A. D. Manchikanti K. Boswell M. Pampati V. Hirsch J. (2015). Efficacy of epidural injections in the treatment of lumbar central spinal stenosis: a systematic review. Anesth. Pain Med. 5, e23139. 10.5812/aapm.23139 25789241 PMC4350165

[B37] Nahm F. S. Lee P. B. Choe G. Y. Lim Y. J. Kim Y. C. (2017). Therapeutic effect of epidural hyaluronic acid in a rat model of foraminal stenosis. J. Pain Res. 10, 241–248. 10.2147/JPR.S122861 28182130 PMC5279814

[B38] Namgoong J. Lee Y. H. Ju A. R. Chai J. Choi D. Choi H. J. (2022). Long-term follow-up of patients with neck pain associated with ossification of the posterior longitudinal ligament treated with integrative complementary and alternative medicine: a retrospective analysis and questionnaire survey. J. Pain Res. 15, 1527–1541. 10.2147/JPR.S356280 35637765 PMC9148200

[B39] Oka H. Matsudaira K. Takano Y. Kasuya D. Niiya M. Tonosu J. (2018). A comparative study of three conservative treatments in patients with lumbar spinal stenosis: lumbar spinal stenosis with acupuncture and physical therapy study (LAP study). BMC Complement. Altern. Med. 18, 19. 10.1186/s12906-018-2087-y 29351748 PMC5775532

[B40] Park S. H. Hong J. Y. Kim W. K. Shin J. S. Lee J. Ha I. H. (2019). Effects of SHINBARO2 on rat models of lumbar spinal stenosis. Mediat. Inflamm. 2019, 7651470. 10.1155/2019/7651470 PMC6512060 31182933

[B41] Renier N. Adams E. L. Kirst C. Wu Z. Azevedo R. Kohl J. (2016). Mapping of brain activity by automated volume analysis of immediate early genes. Cell 165, 1789–1802. 10.1016/j.cell.2016.05.007 27238021 PMC4912438

[B42] Siebert E. Pruss H. Klingebiel R. Failli V. Einhaupl K. M. Schwab J. M. (2009). Lumbar spinal stenosis: syndrome, diagnostics and treatment. Nat. Rev. Neurol. 5, 392–403. 10.1038/nrneurol.2009.90 19578346

[B43] Sondermann J. R. Barry A. M. Jahn O. Michel N. Abdelaziz R. Kugler S. (2019). Vti1b promotes TRPV1 sensitization during inflammatory pain. Pain 160, 508–527. 10.1097/j.pain.0000000000001418 30335684

[B44] Stafford M. A. Peng P. Hill D. A. (2007). Sciatica: a review of history, epidemiology, pathogenesis, and the role of epidural steroid injection in management. Br. J. Anaesth. 99, 461–473. 10.1093/bja/aem238 17704089

[B45] Suto T. Obata H. Tobe M. Oku H. Yokoo H. Nakazato Y. (2012). Long-term effect of epidural injection with sustained-release lidocaine particles in a rat model of postoperative pain. Br. J. Anaesth. 109, 957–967. 10.1093/bja/aes302 22923636

[B46] Vincelette J. Xu Y. Zhang L. N. Schaefer C. J. Vergona R. Sullivan M. E. (2007). Gait analysis in a murine model of collagen-induced arthritis. Arthritis Res. Ther. 9, R123. 10.1186/ar2331 18036238 PMC2246242

[B47] Yang S. Kim W. Kong H. H. Do K. H. Choi K. H. (2020). Epidural steroid injection versus conservative treatment for patients with lumbosacral radicular pain: a meta-analysis of randomized controlled trials. Med. Baltim. 99, e21283. 10.1097/MD.0000000000021283 PMC7386972 32791709

[B48] Zhu J. Ma Y. Xu J. Li Y. Wan P. Qi Y. (2021). Dec-DISCO: decolorization DISCO clearing for seeing through the biological architectures of heme-rich organs. Biomed. Opt. Express 12, 5499–5513. 10.1364/BOE.431397 34692197 PMC8515970

[B49] Zorina-Lichtenwalter K. Parisien M. Diatchenko L. (2018). Genetic studies of human neuropathic pain conditions: a review. Pain 159, 583–594. 10.1097/j.pain.0000000000001099 29240606 PMC5828382

