# Supplementary material for: Repeated epidural delivery of Shinbaro2: effects on neural recovery, inflammation, and pain modulation in a rat model of lumbar spinal stenosis
Source: Front Pharmacol. 2024 May 17;15:1324251. doi: 10.3389/fphar.2024.1324251 (PMC11140021; doi:10.3389/fphar.2024.1324251)
Supplement: Supplementary file 3 [file DataSheet1.docx]

Supplementary Material

#
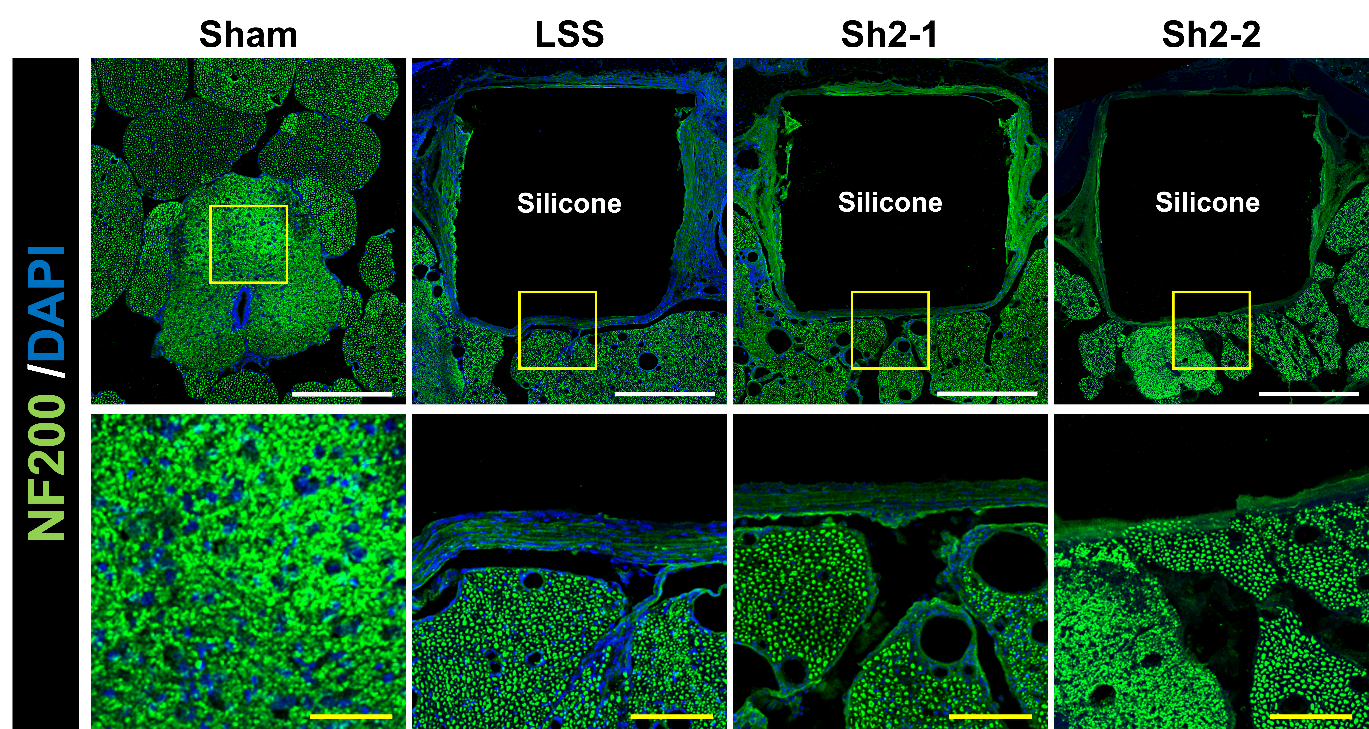


# Supplementary Figure 1. Representative IHC images of NF200 (green) in Sham, LSS, Sh2-1 or Sh2-2 groups. White scale bar = 500 μm, Yellow scale bar =100 μm.
